# Postmastectomy Breast Reconstruction Following Massive Weight Loss: An Updated Systematic Review and Identification of Research Gaps

**DOI:** 10.1002/micr.70185

**Published:** 2026-01-30

**Authors:** Anna Paganini, Jonas Löfstrand, Nushin Mirzaei, Emma Hansson

**Affiliations:** ^1^ Department of Plastic Surgery, Institute of Clinical Sciences, The Sahlgrenska Academy University of Gothenburg Gothenburg Sweden; ^2^ Department of Plastic Surgery Sahlgrenska University Hospital, Region Västra Götaland Gothenburg Sweden; ^3^ Department of Diagnostics, Acute and Critical Care, Institute of Health and Care Sciences, Sahlgrenska Academy at the University of Gothenburg University of Gothenburg Gothenburg Sweden; ^4^ Department of Surgery, Sahlgrenska Centre for Cancer Research, Institute of Clinical Sciences, Sahlgrenska Academy University of Gothenburg Gothenburg Sweden

**Keywords:** breast cancer, breast reconstruction, massive weight loss, post‐bariatric reconstruction, risk factors for complications

## Abstract

**Background:**

As bariatric surgery becomes increasingly common, reconstructive surgeons are encountering more massive weight loss (MWL) patients requiring breast reconstruction. MWL alters breast anatomy, tissue characteristics, and healing capacity, potentially impacting reconstructive outcomes.

**Objective:**

To update and evaluate the evidence on how MWL affects complication and revision rates, flap‐relevant anatomy, and patient‐reported outcomes (PROMs) following postmastectomy breast reconstruction. In addition, research gaps were identified.

**Methods:**

This systematic review updated a previous review and followed PRISMA guidelines. Eligible studies included cohort studies and case series of postmastectomy breast reconstruction after MWL. Data extraction and appraisal were performed independently by two reviewers, with evidence quality rated using the GRADE system.

**Results:**

Fifteen studies met the inclusion criteria, including three case–control studies and twelve case series, reporting outcomes for 102 patients after massive weight loss (MWL). Most reconstructions used abdominally based free flaps, with few MWL‐specific modifications. MWL patients experienced significantly higher rates of delayed wound healing, surgical‐site infection, fat necrosis, and need for revision compared with controls, while total flap loss rates were similar. Evidence on implant‐based reconstruction, vascular anatomy, and PROMs was scarce. The overall certainty of evidence was very low (GRADE ⊕⊝⊝⊝).

**Conclusion:**

Breast reconstruction after MWL is associated with increased wound‐healing complications and revision rates, though patient satisfaction appears acceptable. Evidence remains limited by small, heterogeneous studies and a lack of controlled or prospective data. Future research should address optimal reconstructive techniques, timing, and patient selection, including the identification of modifiable risk factors and the use of PROMs to guide evidence‐based care.

**Abbreviations**
BMI: body mass index
CI: confidence interval
DIEP: deep inferior epigastric perforator (flap)
DUG: diagonal upper gracilis (flap)
GAP: gluteal artery perforator (flap)
IMAP: internal mammary artery perforator (flap)
LD: latissimus dorsi (flap)
MS: muscle sparing
MWL: massive weight loss
*N*
: number
NA: not applicable
NR: not reported
OR: odds ratio
PAP: profunda artery perforator (flap)
SD: standard deviation
SEAP: superficial external abdominal perforator (flap)
SIEA: superficial inferior epigastric artery (flap)
TDAP: thoracodorsal artery perforator (flap)
TRAM: transverse rectus abdominis myocutaneous (flap)
TUG: transverse upper gracilis (flap)
V: vertical
VMG: vertical gracilis myocutaneous flap

## 1 Introduction

Bariatric surgery is common; between 2008 and 2018, 6.5 million people underwent bariatric surgery globally (Lazzati 2023). As breast cancer is the most common cancer in women, the number of post‐bariatric patients diagnosed with breast cancer is considerable (Doumouras et al. 2023). In addition, bariatric surgery has been suggested as a bridge therapy to decrease the surgical risks for patients with obesity who want a breast reconstruction (Hammond et al. 2025). This means that reconstructive surgeons will face increasing numbers of massive weight loss (MWL) patients in need of breast reconstruction.

The MWL following bariatric surgery includes anatomical breast alterations, such as a reduction in volume, an increased sternal notch–to–nipple distance, accentuated ptosis, inferior–medial displacement of the nipple–areolar complex, redundant skin, attenuation of the lateral breast contour, and weakening of the inframammary fold (Agha‐Mohammadi and Hurwitz 2008b; Albino et al. 2009; Beidas and Gusenoff 2019). Significant weight loss can also modify the breast's internal architecture by changing tissue density, the proportion of fibroglandular to total breast volume (Sun et al. 2023), and the number of elastic fibers (Hany et al. 2024), factors which all influence skin strength and resilience. In addition, post‐bariatric patients potentially have nutritional deficiencies and compromised wound healing (Agha‐Mohammadi and Hurwitz 2008b; Albino et al. 2009; Beidas and Gusenoff 2019). These factors might necessitate adapted breast reconstruction techniques to achieve acceptable results with low complication risk.

L. Sinik et al. (2022) conducted a systematic review of studies published in March 2020 or earlier on breast reconstruction in patients who have undergone MWL with the aim of summarizing surgical techniques used in MWL patients. A total of 497 articles were screened, with 10 meeting the inclusion criteria. These were analyzed for surgical indications, advantages, disadvantages, complications, and outcomes related to breast reconstruction in patients with a history of MWL. The overall level of evidence was low, since the included articles were primarily retrospective cohort studies, case series, and case reports. As the patient population is growing and 6 years have passed since the latest systematic review, an updated review is warranted.

The present updated systematic review primarily aimed to investigate the evidence for the effect of MWL on complication and revision rates, flap‐relevant anatomy, and patient‐reported outcomes (PROMs) in breast reconstruction after mastectomy. A secondary aim of the review was to identify and discuss any related research gaps in need of further studies. In relation to previous reviews, the present article includes studies published over the past 6 years and provides evidence grading for different outcomes.

## 2 Methods

This is an update of a systematic review performed by L. Sinik et al. (2022), who searched articles through March 2020. Results were reported according to the Preferred Reporting Items for Systematic Reviews and Meta‐Analyses (PRISMA) guidelines (Page et al. 2021). Inclusion criteria were cohort studies and case series examining postmastectomy breast reconstruction following MWL. Narrative review articles, comments, technical descriptions, communications, study protocols, and editorials were excluded. Included articles had to meet criteria defined in a PICO (Patients, Intervention, Comparison, and Outcome of interest), based on the previous review (L. Sinik et al. 2022). P: patients having postmastectomy breast reconstruction following MWL, I: postmastectomy breast reconstruction with any technique, C: patients having postmastectomy breast reconstruction who have not had MWL, O1: complications, O2: revisions, O3: vessel anatomy, O4: PROMs. MWL, postmastectomy breast reconstruction, immediate versus delayed breast reconstruction, complications, and thresholds for complications were not predefined before the search. Three authors (A.P., N.M., and E.H.) independently assessed if the articles met the inclusion criteria, and disagreements were resolved through discussion among authors. The PubMed, Embase, and Cochrane Central Register of Controlled Trials were searched, as in the original review, for articles published from January 2020 to September 2025. The search was conducted on September 30, 2025. The search string was (breast reconstruction OR mastectomy) AND (massive weight loss OR bariatric surgery), as in the original review (L. Sinik et al. 2022). The search was limited to studies published in English. Full articles were assessed when eligibility for inclusion could not be evaluated based on the abstract alone. Data were extracted independently by two authors (A.P. and E.H.) in duplicate. Any disagreements were resolved through discussion. Information collected included: all variables included in the previous review (L. Sinik et al. 2022), study country, follow‐up time, and the result of any comparisons between patients and controls. Information on how the studies defined MWL, postmastectomy breast reconstruction, immediate versus delayed breast reconstruction, complications, and thresholds for complications was also extracted. The results of each article were tabulated per outcome (Table 1). No efforts were made to obtain additional data or confirm the data in the articles from the original investigators. As the studies used different definitions of complications, data were not pooled. The actual numbers of complications reported in the original publications are presented in the Tables. In addition, complication and revision frequencies were calculated for case series of 30 or more breasts. The authors did not consider complication frequencies in case series of fewer than 30 cases to be meaningful.

**TABLE 1:** Included studies.

Author, year, country	Study design (*main scope*)	Follow‐up (months)	Type of breast reconstruction (no. patients/no. flaps)	Included patients (*n*) (included breasts, *n*)	Included controls (*n*) (included breasts, *n*) type of breast reconstruction	Outcome variables
Abdel‐Naby, 2017, USA^a^ (Abdel‐Naby et al. 2017; L. Sinik et al. 2022)	Case report	2	Autologous Pedicled TRAM (1/1)	1 (1)	NA	O1: Complications
Asiry, 2019, France^a^ (Asiry et al. 2019; L. Sinik et al. 2022)	Case report	12	Reverse abdominoplasty advancement + lipofilling	1	NA	O1: Complications O2: Revisions
Bauder, 2018, USA^a^ (Bauder et al. 2018; L. Sinik et al. 2022)	Case control	6	Autologous DIEP (NR/4) SIEA (NR/5) TRAM (NR/14) TUG (NR/3)	14 (26)	1012 (1572) DIEP (NR/373) GAP (NR/20) SIEA (NR/102) TRAM (NR/1053) TUG (NR/18)	O1: Complications O2: Revisions
Berkane 2024, USA (Berkane et al. 2024)	Caser report	6	Autologous Fleur‐de‐lis DIEP	1 (1)	0	O1: Complications O2: Revisions
Chakari 2024, Denmark (Chakari et al. 2024)	Case report	NR	Autologous Stacked IMAP + TDAP (1/2) Stacked TDAP + SEAP (1/2)	2^b^ (4)	0	O2: Revisions
Cogliandro, 2018, Italy^a^ (Cogliandro et al. 2018; L. Sinik et al. 2022)	Case series	12 (range 4–36)	Implant‐based	20 (28)	NA	O1: Complications
Dayicioglu, 2016, USA^a^ (Dayicioglu et al. 2016; L Sinik et al. 2022)		NR	Autologous DIEP (6/9)	6 (9)	18 (Selected among 97 controls) DIEP	O1: Complications
Gusenoff, 2008, USA^a^ (Gusenoff et al. 2008; L. Sinik et al. 2022)	Anatomy study	NR	Breast reconstruction not performed	32 (64 hemiabdomen)	NA	O3: Vessel anatomy
Gusenoff, 2009, USA^a^ (Gusenoff et al. 2009) (L. Sinik et al. 2022)	Case report	18	Autologous TRAM (1/4) DIEP (1/1)	3 (5)	NA	O1: Complications
Martinez, 2016, USA (Martinez et al. 2016; L. Sinik et al. 2022)	Case series	16.2 (range 3.5–28.7)	Autologous DIEP (9/18) 13 immediate and 5 delayed cases	9 (18)	NA	O1: Complications O2: Revisions
Salim, 2013, UK^a^ (Salim et al. 2013; L. Sinik et al. 2022)	Case report	6	Autologous Fleur‐de‐lis DIEP (1/1)	1 (1)	NA	O1: Complications O2: Revisions
Sinik, 2021, USA (L. M. Sinik et al. 2023)	Case–control	6	Autologous SIEA (NR/2) DIEP (NR/60) PAP (NR/2) TUG/DUG (NR/4)	39 (68)	877 (139) SIEA (NR/39) DIEP (NR/90) MS‐TRAM (NR/14) PAP (NR/15) TUG/DUG (NR/75)	O1: Complications O2: Revisions O4: Patient reported outcomes (PROMs)
Söderman, 2021, Denmark (Soderman et al. 2021)		3	Auotlogous VMG (1/1)	1 (1)	NA	O1: Complications O2: Revisions
Wechselberger, 2000, Switzerland^a^ (Wechselberger et al. 2000) (L. Sinik et al. 2022)		NR	Autologous DIEP (1/2)	1 (2)	NA	O1: Complications O2: Revisions
Yoo, 2022, USA (Yoo et al. 2022)		7	Autologous Stacked DIEP + vPAP (2/4)	2 (4)	NA	O1: Complications O2: Revisions

^a^ Article included in the original review.

^b^ The authors state they have operated on 10 patients, but only report data for 2 patients.

Risk of bias was assessed for case–control studies using the Risk Of Bias In Non‐randomized studies—of Interventions (ROBINS‐I) tool (Schunemann et al. 2019). ROBINS‐I includes seven domains of bias: due to confounding, classification of interventions, selection of participants into the study, missing data, measurement of the outcome, deviation from the intended protocol, and selection of the reported results. The risk of bias assessment has four levels: low, moderate, serious, and critical. In addition, an overall risk‐of‐bias judgment is generated from the domain assessments using an algorithm (Schunemann et al. 2019). Risk of bias was not assessed for case series/reports.

The overall certainty of evidence for different outcomes was classified as very low (GRADE ⊕⊝⊝⊝), low (GRADE ⊕ ⊕ ⊝⊝), moderate (GRADE ⊕ ⊕ ⊕⊝), or high (GRADE ⊕ ⊕ ⊕⊕) according to the GRADE system (Grading of Recommendations, Assessment, Development and Evaluations) (Guyatt et al. 2008). Anatomical studies were not appraised.

## 3 Results

### 3.1 Included Studies, Cases, and Controls

**FIGURE 1:**
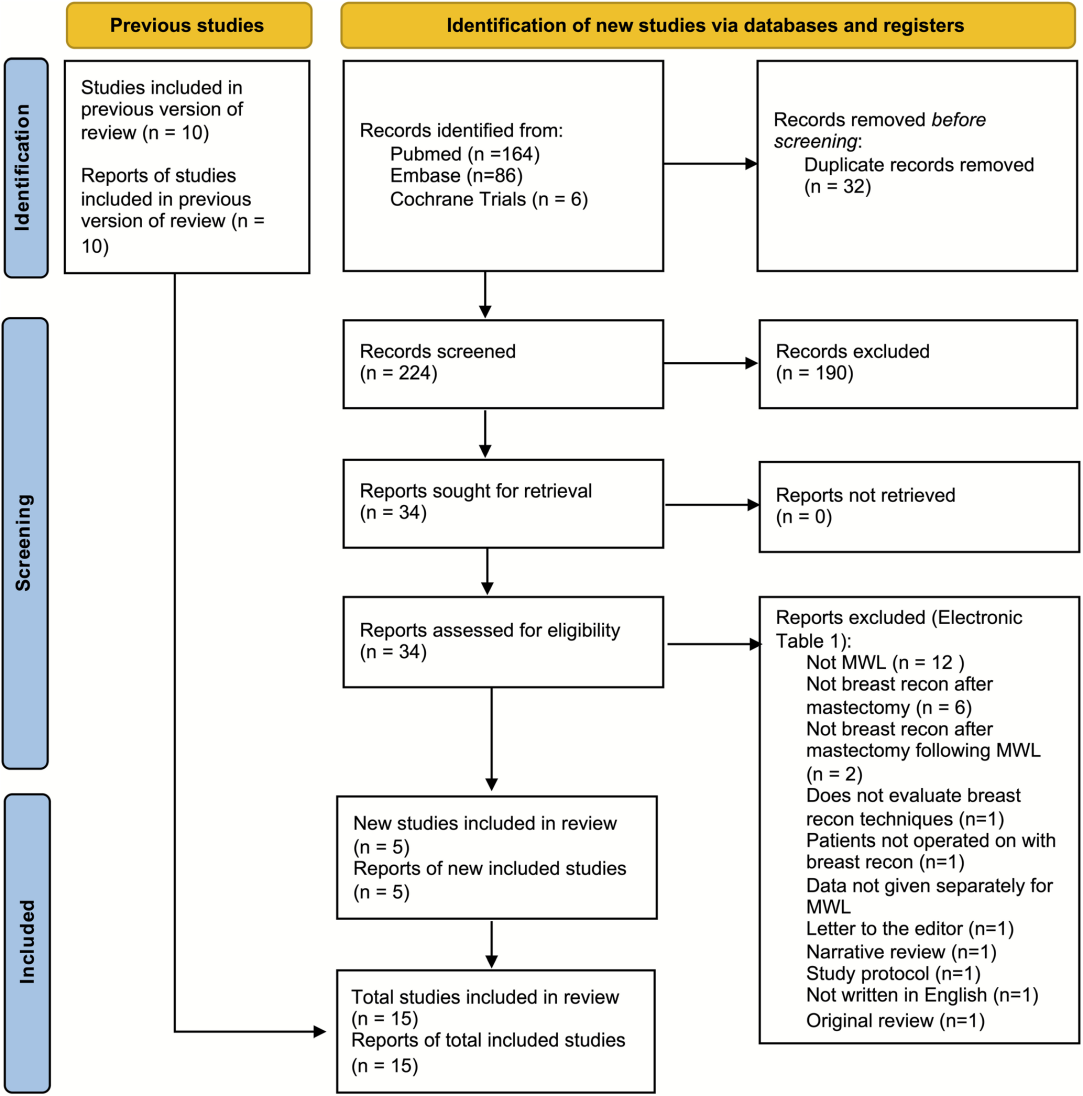
PRISMA diagram. Breast recon, breast reconstruction; MWL, massive weight loss. *Source:* Page et al. (2021).

The 10 studies included in the original review were included. The new searches retrieved a total of 224 abstracts after duplicates had been removed. Of these, 190 did not meet the inclusion criteria. After full texts had been read, a further 29 manuscripts could be excluded (Supporting Information 1), leaving six studies to be included in the review. There were no discrepancies between the two authors who selected the articles, so no discussion regarding inclusion was required. This resulted in a total of 15 studies to include in the review (Figure 1, Table 1). Among the included studies, there were three case–control studies, including 6–39 cases and 18–1012 controls, and 12 case reports/series. In total, data are reported for 102 patients who have experienced MWL (Table 1). Details on how the different studies defined MWL, weight stability, postmastectomy breast reconstruction, immediate versus delayed reconstructions, different complications, and thresholds for complications are given in Supporting Information 2.

### 3.2 Risk of Bias of the Included Case–Control Studies

Risk‐of‐bias assessment using the ROBINS‐I tool demonstrated that all three included case–control studies were at moderate to serious overall risk of bias. The primary source of bias across studies was confounding, consistently judged as *serious*, reflecting the retrospective study designs, imbalance in baseline characteristics between MWL and control cohorts, and limited adjustment methods. Selection bias was judged *moderate* in all studies due to retrospective case identification and potential surgeon‐ or patient‐related factors influencing inclusion. Across studies, classification of interventions was considered *low risk*, as MWL status and reconstruction type were objectively defined and reliably extracted from clinical records. Deviations from intended interventions were generally *moderate* because perioperative management was not standardized, although no evidence suggested systematic differences in care. Missing data ranged from *moderate to serious*, driven by incomplete follow‐up, undocumented minor complications, and loss to follow‐up for PROMs. Outcome measurement bias ranged from *moderate to serious*, as several outcomes (e.g., delayed wound healing, fat necrosis) rely on clinician judgment without blinded assessment. Moreover, only one study defined the complications. Finally, selective reporting was judged *moderate* across all studies due to the absence of preregistered protocols and potential bias toward reporting statistically significant findings. Overall, the risk‐of‐bias profiles indicate that the current evidence base is limited by methodological constraints inherent to retrospective observational designs. Details on the ROBINS‐I evaluations are given in Supporting Information 3.

### 3.3 Development of Surgical Techniques Since the Previous Review

Three case–control studies and 10 case reports/series reported on autologous reconstruction. The great majority of reported flaps were abdominally based free flaps (*n* = 121) (Table 1). The only described MWL‐specific modification in these techniques is the fleur de lis deep inferior epigastric perforator flap (DIEP), “the 5th DIEP zone.” The modification was described before the last review was published (Salim et al. 2013), and only one additional case has been published since then (Berkane et al. 2024). A limited number of thigh‐based flaps (*n* = 7) have been reported (Table 1). Since the previous review, the concept of stacked free flaps has been popularized (Haddock and Teotia 2023), and four stacked flaps (eight individual flaps in four patients) have been reported.

Regarding other techniques than free flaps, one case series report on 28 implant‐based breast reconstructions in 20 MWL patients (Cogliandro et al. 2018), one report on one case of reverse abdominoplasty advancement in combination with lipofilling (Asiry et al. 2019), and one pedicled transverse rectus myocutaneous flap (TRAM) (Abdel‐Naby et al. 2017). All these publications were included in the previous review.

### 3.4 Complications and Revisions (O1 and O2)

Fourteen of the 15 included studies reported on complications and/or revision procedures (Table 1). Across studies, complication rates varied widely, reflecting differences in reconstructive technique, reporting standards, and patient selection. Details about the reported data are given in Supporting Information 4.

Among the three case–control studies, L. M. Sinik et al. (2023), comparing 39 patients and 877 controls, provided the most detailed comparative data. MWL patients experienced significantly higher rates of delayed wound healing both at the flap site (23.5% vs. 11.7%, *p* = 0.003) and donor site (43.6% vs. 27.6%, *p* = 0.03), as well as higher rates of surgical‐site infection (7.4% vs. 2.2%, *p* = 0.02), fat necrosis (25% vs. 16%, *p* = 0.05), and partial flap loss (5.9% vs. 1.6%, *p* = 0.03) compared to controls. No significant differences were observed for thrombosis, congestion, or total flap loss. The rate of blood transfusion was significantly higher in the MWL group (25.6% vs. 9.9%, *p* = 0.005). The same study also reported a significantly higher number of incisional flap and donor‐site revisions in MWL patients (*p* = 0.009 and *p* = 0.01, respectively). In contrast, the number of fat‐grafting revisions and total fat graft volume were similar between groups. Bauder et al. (2018), comparing 14 patients and 1012 controls, found a higher rate of delayed breast healing in MWL patients (57% vs. 34%), although this did not reach statistical significance (*p* = 0.087). The mean number of breast revision procedures was significantly greater in MWL patients compared to controls (1.35 vs. 0.61; *p* = 0.0055), as was the number of implant or expander placements (*p* = 0.0003). Dayicioglu et al. (2016), comparing 6 patients and 18 controls, reported similar rates of flap loss and donor‐site morbidity between the MWL and control groups. However, their sample size was small (6 MWL and 18 controls), and only complications requiring surgical intervention were recorded.

Across case series and case reports, reported complications were generally minor and procedure‐specific. Martinez et al. (2016) observed one breast abscess and one abdominal suture cyst in nine DIEP reconstructions. Single‐case reports described isolated complications such as donor‐site hematoma (Soderman et al. 2021), abdominal seroma (Salim et al. 2013), abdominal fat necrosis (Yoo et al. 2022), or donor‐site bulging (Gusenoff et al. 2009). No flap losses were reported in any case report.

The only study on implant‐based breast reconstruction reported one wound dehiscence and one case of asymmetry among 20 cases.

Overall, the pooled evidence indicates that MWL patients undergoing autologous reconstruction experience higher rates of wound‐healing complications and need for secondary revision procedures compared to non‐MWL controls. However, the specific rates of different complications and revisions remain unclear, as only a small, limited series of patients has been published. The majority of included studies are small retrospective case series and case reports with heterogeneous methodologies, definitions of complications, and short follow‐up periods. Only three comparative (case–control) studies exist, and these display substantial variation in surgical techniques, outcome reporting, and control group matching. Moreover, limited demographic data are provided for the patients, and analyses are not corrected for any synchronous risk factors.

The overall certainty of evidence for the effect of MWL on risk and revision after autologous breast reconstruction is very low (GRADE ⊕⊝⊝⊝). The evidence was downgraded three levels due to a very high risk of bias, indirectness, and imprecision. No evidence exists regarding the effect of MWL on risk and revision after implant‐based breast reconstruction.

### 3.5 Vessel Anatomy (O3)

One study reported vessel anatomy (Gusenoff et al. 2008) (Table 1). The survey of 32 SIEAs found a significant association between the highest recorded BMI and the identification of a SIEA (*p* = 0.009) and the likelihood of having a usable artery (*p* = 0.04). Both current and maximum BMI were significantly linked to the diameter of the SIEV (*p* = 0.001).

### 3.6 PROMs (O4)

One study, comparing 39 MWL patients and 877 controls, reported PROMs (L. M. Sinik et al. 2023). Ten MWL patients (response rate 26%) and 187 controls (response rate 21%) answered BREAST‐Q 6 months postoperatively. Preoperative scores were not available. The study found that MWL patients had lower psychosocial well‐being (53.5 ± 20.4 vs. 71.3 ± 19.5 [*p* = 0.01]) and lower sexual well‐being (37.7 ± 17 vs. 52.1 ± 23.1 [*p* = 0.01]) than the controls. MWL patients also had lower scores for satisfaction with breast/s (54 ± 19 vs. 62.4 ± 12.2 [*p* = 0.18]) and physical well‐being chest (69.8 ± 20.8 vs. 76.6 ± 18.1 [*p* = 0.44]), although the difference was not statistically significant. No analyses were performed regarding differences between responders and non‐responders or adjustment for potential confounders (L. M. Sinik et al. 2023). The overall certainty of evidence for the effect of MWL on PROMs after breast reconstruction is very low (GRADE ⊕⊝⊝⊝). The evidence was downgraded three levels due to a high risk of bias, indirectness, imprecision, and publication bias.

### 3.7 Research Gaps

**FIGURE 2:**
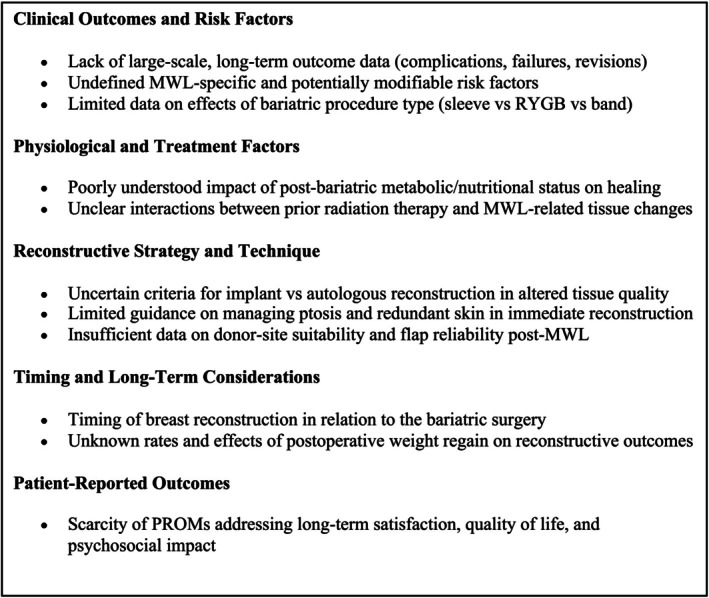
Research gaps regarding postmastectomy breast reconstruction in patients with previous massive weight loss.

In patients following MWL, the current evidence‐based guiding breast reconstruction remains limited. The identified research gaps are elaborated upon in Section 4 and summarized in Figure 2.

## 4 Discussion

This updated systematic review demonstrates that evidence regarding the outcomes of postmastectomy breast reconstruction in patients following MWL remains scarce and of low methodological quality. Despite an increasing clinical need, only a small number of studies with limited sample sizes and heterogeneous designs have been published since the last systematic review by L. Sinik et al. (2022). The newly identified studies strengthen the conclusions of the previous review (L. Sinik et al. 2022) that MWL patients appear to experience higher rates of wound‐healing‐related complications and require more revisional procedures than non‐MWL controls. It is also in accordance with the previously identified higher risk for complications after body contouring surgery in the population (Marouf and Mortada 2021; L. M. Sinik et al. 2023). Nonetheless, the wide variation in definitions, reporting standards, and follow‐up durations across included studies limits the ability to draw firm conclusions about the extent of the risk in the MWL population. Moreover, the absence of prospective, adequately powered studies introduces a high risk of bias, and none of the included reports adjusted for potential confounders such as age, smoking status, comorbidities, or adjuvant therapies. Another limitation of the included studies is that they have all been performed in high‐income countries with microsurgical expertise, limiting their applicability in low‐resource settings. The certainty of this evidence remains very low, and it is still unknown how much the risks increase and what specific risk factors there are.

Since the prior review, few new technical innovations have been described specifically for the MWL population. The fleur‐de‐lis DIEP modification remains the only MWL‐adapted approach reported in the literature (Berkane et al. 2024; Salim et al. 2013). Although the advent of stacked free‐flap techniques (Haddock and Teotia 2023) may expand reconstructive options for patients with limited donor tissue, evidence of their efficacy and safety in the MWL population remains anecdotal. Limited data exist on the influence of MWL on vascular anatomy relevant to flap planning. One anatomical study found associations between body mass index (BMI) and superficial inferior epigastric artery (SIEA) presence and caliber (Gusenoff et al. 2008), suggesting that prior obesity and weight loss may alter vascular patterns. These findings require validation and correlation with clinical outcomes before influencing surgical planning and flap selection.

It has been proposed that autologous breast reconstruction represents an optimal approach for breast reconstruction in the MWL population, as it utilizes redundant soft tissue that patients typically seek to have excised as part of their post‐weight loss body contouring procedures (Dayicioglu et al. 2016; Wechselberger et al. 2000; Yoo et al. 2022). However, autologous reconstruction is not appropriate for all patients due to underlying medical conditions. In addition, some patients who have undergone MWL may have previously received body contouring procedures before the diagnosis of breast cancer, thereby limiting the availability of suitable donor sites. This necessitates that other reconstructive techniques be studied in the population. Data on implant‐based reconstruction are minimal, as only one case series exists (Cogliandro et al. 2018). It is well known that the characteristic alterations in skin redundancy, ptosis, and tissue elasticity following MWL might necessitate modified techniques regarding management of the skin envelope and ptosis and implant stabilization, in benign breast reconstructions, such as augmentation‐mastopexy (Coombs et al. 2017; Junior et al. 2016; Motamedi et al. 2024; Salgarello and Visconti 2020; Vindigni et al. 2015). In an oncological setting, there might be additional factors affecting the prerequisites, such as radiotherapy. The effect of radiotherapy on post‐bariatric tissue quality has never been studied. Further studies are needed to determine the optimal reconstructive strategy and how the reconstructive techniques can be modified to suit the prerequisites of MWL patients.

None of the published studies have studied risk factors for complications and revision in the MWL population. The physiological consequences of MWL and bariatric surgery may contribute to the elevated complication profile observed in this systematic review. Nutritional deficiencies are well‐documented after significant weight loss and may impair wound healing (Agha‐Mohammadi and Hurwitz 2008b). However, none of the included studies systematically assessed nutritional or metabolic parameters in relation to surgical outcomes. Similarly, the impact of the type of prior bariatric procedure (e.g., sleeve gastrectomy versus Roux‐en‐Y gastric bypass) on breast reconstruction remains unexplored (Kwon et al. 2022). Another important factor identified in body contouring procedures and benign breast reconstruction in the MWL population is timing, specifically, determining the optimal interval between bariatric surgery and plastic surgery to minimize postoperative complications (Hansson et al. 2025) (Agha‐Mohammadi and Hurwitz 2008a, 2008b; Albino et al. 2009; Beidas and Gusenoff 2019; Hurwitz and Golla 2004; Marouf and Mortada 2021). Optimal timing of postmastectomy breast reconstruction in the MWL population has never been studied and might affect the selection between immediate and delayed breast reconstruction. The factors described above may play essential roles in reconstructive success but are underrepresented in current research.

A breast reconstruction is ultimately performed to increase a patient's quality of life, which makes PROMs an essential outcomes measure (Wormald and Rodrigues 2018). The only study including PROMs reports data from 10 MWL patients (L. M. Sinik et al. 2023). Thus, very little is known about PROMs in after breast reconstruction in this population. Studies comparing PROMs between different techniques, different timings, and long‐term are needed to optimize reconstruction in the population.

The present review has several limitations. First, the original search string from a previously published review (L. Sinik et al. 2022) was used: (breast reconstruction OR mastectomy) AND (massive weight loss OR bariatric surgery). The search string is very simplistic, includes few synonyms, no MeSH/Emtree terms, and no alternative spellings, which might have affected the search results. However, the broad nature of the search term should not have yielded a high miss rate; rather, it should have yielded too many irrelevant hits. Second, the search was limited to studies published in English. However, when the search is made without any language limitations, only five additional studies, which do not include postmastectomy breast reconstruction (Flandroy et al. 2023; Goldammer et al. 2020; Ngo et al. 2022; Thomsen et al. 2022; Yang et al. 2020) are found. This indicates that the restriction on publication language has not led to biased study selection. Thirdly, it can be discussed whether the data from the three case–control studies should have been pooled. However, because the patient populations, surgical techniques, and outcome definitions across the three studies were heterogeneous, it was determined that the studies' similarities and data quality were insufficient, and therefore, pooling and meta‐analysis were not considered scientifically sound.

In conclusion, this updated systematic review highlights the paucity of high‐quality evidence on breast reconstruction after MWL. The included observational retrospective studies have a high risk of bias. The certainty of this evidence remains very low for complications, revisions, anatomical considerations, and PROMs. The studies point in the direction that there is an elevated risk of complications and secondary revisions in patients who have had MWL before their breast reconstruction. However, it is still unknown by how much the risks increase and which specific risk factors are involved. Several research gaps have been identified (Figure 2).

## Funding

The study was funded by grants from the Swedish Cancer Society (210279 SCIA) and the federal government under the ALF agreement (ALFGBG‐1005048). The sources of funding had no role in the study's design, data collection, analysis, interpretation, or manuscript writing.

## Conflicts of Interest

The authors declare no conflicts of interest.

## Supporting information


**Supporting Information: 1.** Studies excluded after reading the full text.

**Supporting Information: 2.** Definitions of the included articles.

**Supporting Information: 3.** ROBINS‐I evaluation of included case‐ control studies.

**Supporting Information: 4.** Complications and revisions (O1 and O2).

## Data Availability

The data that supports the findings of this study are available in the Supporting Information of this article.
