# Supplementary material for: Postmastectomy Breast Reconstruction Following Massive Weight Loss: An Updated Systematic Review and Identification of Research Gaps
Source: Microsurgery. 2026 Jan 30;46(2):e70185. doi: 10.1002/micr.70185 (PMC12856973; doi:10.1002/micr.70185)
Supplement: Supplementary file 1 — Supporting Information: 1. Studies excluded after reading the full text. [file MICR-46-e70185-s001.docx]

# Electronic supplement 1: Studies excluded after reading the full text

| **Study (first author)** | **Reason for exclusion** |
| --- | --- |
| Abboud (M. H. Abboud, El Hajj, Kapila, Bogaert, & Abboud, 2022) | Not MWL |
| Abboud (N. M. Abboud, Hajj, & Abboud, 2021) | Not MWL |
| Atiyeh (Atiyeh & Chahine, 2022) | Not MWL |
| Cason (Cason et al., 2020) | Not MWL |
| Krishnammoorthy (F. Chen et al., 2020) | Not breast reconstruction after mastectomy |
| Chen (Z. Chen et al., 2025) | Not written in English |
| Cintra Junior (Cintra Junior, Modolin, Colferai, Rocha, & Gemperli, 2021) | Not breast reconstruction after mastectomy |
| De Macedo (de Macedo, Rosa, Canedo, & Casulari, 2020) | Not breast reconstruction after mastectomy |
| Dolis Marretto de Moura (Dolis Marretto de Moura et al., 2025) | Not breast reconstruction after mastectomy |
| Eckhoff (Eckhoff et al., 2022) | Not MWL |
| Fernandes (Fernandes et al., 2023) | Does not evaluate reconstructive techniques |
| Hagiga (Hagiga et al., 2022) | Not MWL |
| Hammond (Hammond, Mathes, Winocour, & Kaoutzanis, 2024) | Letter to the editor |
| Hammond (Hammond et al., 2025) | Patients not operated with breast reconstruction, simulation, risk calculation model |
| Kotsougiani-Fischer (Kotsougiani-Fischer et al., 2021) | Not MWL |
| Lee (Lee & Park, 2022) | Not breast reconstruction after mastectomy |
| Lipman (Lipman, Graw, & Nguyen, 2021) | Not breast reconstruction after mastectomy after MWL |
| Papas (Papas, Bou-Merhi, Odobescu, Retchkiman, & Danino, 2021) | Not MWL |
| Pinto (Pinto, Pignatti, Contu, & Cipriani, 2022) | Not MWL |
| Pompei (Pompei, Abate, & Guido, 2020) | Not breast reconstruction after mastectomy after MWL |
| Rampazzo (Rampazzo et al., 2023) | Data not given separately for patients who have had MWL |
| Renom (Renom et al., 2024) | Not MWL |
| Shiraishi (Shiraishi et al., 2022) | Not MWL |
| Siegwart (Siegwart et al., 2022) | Not MWL |
| Sinik (L. Sinik, Egan, Patel, Nazir, & Butterworth, 2022) | *Original review, already included* |
| Sinik (L. M. Sinik & Collins, 2024) | Narrative review |
| Thornton (Thornton, Edalatpour, & Gast, 2024) | Not breast reconstruction after mastectomy |
| Varnava (Varnava et al., 2023) | Data not given separately for patients who have had MWL |
| RBR (y4bvbw, 2021) | Study protocol |
