# Supplementary material for: Postmastectomy Breast Reconstruction Following Massive Weight Loss: An Updated Systematic Review and Identification of Research Gaps
Source: Microsurgery. 2026 Jan 30;46(2):e70185. doi: 10.1002/micr.70185 (PMC12856973; doi:10.1002/micr.70185)
Supplement: Supplementary file 2 — Supporting Information: 2. Definitions of the included articles. [file MICR-46-e70185-s003.docx]

# Electronic supplement 2: Definitions of the included articles

| **Author, Year, Country** | **Definition of MWL** | **Definition of weight stability** | **Definition of post-mastectomy breast reconstruction**  **Immediate vs. delayed breast reconstruction** | **Definition of complications and thresholds for complications** |
| --- | --- | --- | --- | --- |
| Abdel-Naby, 2017, USA* (Abdel-Naby, Ablavsky, & Shteynberg, 2017) | Following sleeve gastrectomy, the individual achieved a 105-pound weight reduction | 12 months | Delayed breast reconstruction following a mastectomy 8 months earlier. | NR |
| Asiry, 2019, France* (Asiry, Garrido, Chaput, Chantalat, & Vaysse, 2019) | Following gastric by-pass, three years ago, the patient lost 64 kg | NR | Delayed breast reconstruction following a mastectomy 2 years earlier. | NR |
| Bauder, 2018, USA* (Bauder et al., 2018) | Weight loss following a bariatric operation (including open or laparoscopic gastric banding, roux-en-y bypass, or sleeve gastrectomy). | >6 months | NR | Delayed wound healing at the breast or abdominal donor site: skin necrosis or wound breakdown requiring topical care or dressing changes for more than 3 weeks.  Fat necrosis: a palpable area of firmness greater than 1 cm on clinical examination during follow-up, not attributable to cancer  Surgical revisions, hematoma, seroma, delayed reconstructive failure: Complications not defined |
| Berkane, 2024, USA (Berkane et al., 2024) | The patient had a gastric band placed and, six years later, proceeded with a gastric bypass. Together, these interventions produced a 41-kg weight loss and lowered her BMI from 42.9 to 27.9 kg/m² | NR | Immediate-delayed:  An expander was placed at the same time as the mastectomy. 12 months later the expander was exchanged for a DIEP-flap. | NR |
| Chakari, 2024, Denmark (Chakari, Bille, Lilja, & Thomsen, 2024) | NR | NR | NR | NR |
| Cogliandro, 2018, Italy* (Cogliandro, Barone, Cassotta, Salzillo, & Persichetti, 2018) | NR | NR | NR | NR |
| Dayicioglu, 2016, USA* (Dayicioglu et al., 2016) | >30 kg weight loss | NR | NR | NR |
| Gusenoff, 2008, USA* (Gusenoff, Coon, De La Cruz, & Rubin, 2008) | NR | NA | NA | NA |
| Gusenoff, 2009, USA*(Gusenoff et al., 2009) | Weight loss following bariatric surgery | NR | NR | NR |
| Martinez, 2016, USA (Martinez, Walters, Sato, Hall, & Boutros, 2016) | Weight loss following bariatric surgery | NR | NR | NR |
| Salim, 2013, UK* (Salim, Adlard, & Pickford, 2013) | Weight loss following bariatric surgery | NR | Delayed breast reconstruction performed several years after mastectomy | NR |
| Sinik, 2021, USA (Sinik et al., 2023) | A history of bariatric surgery or weight loss >50 pounds | NR |  | Arterial compromise – change in physical examination or return to theatre  Venous congestion – changes in physical examination or or return to theathre  Partial flap loss – less than 100% of a flap required debridement  Delayed wound healing – “notation of wound or eschar on examination or recording local wound care, procedures or operative interventions for delayed wound healing”  Surgical-site infection – “change in physical examination requiring enteral or parental antibiotics”  Seroma – requiring intervention with aspiration, drain replacement or operation |
| Söderman, 2021, Denmark (Soderman, Thomsen, & Sorensen, 2021) | Bariatric surgery  Reduction from BMI 47 to 31 | NR | Delayed breast reconstruction performed several years after mastectomy | NR |
| Wechselberger, 2000, Switzerland* (Wechselberger, Haug, Schoeller, Nehoda, & Piza-Katzer, 2000) | Vertical banded gastroplasty  Reduction from BMI 52 to 26 | NR | NR | NR |
| Yoo, 2022, USA (Yoo et al., 2022) | Gastric bypass  -44% of body weight | NR | NR | NR |
