# Supplementary material for: Postmastectomy Breast Reconstruction Following Massive Weight Loss: An Updated Systematic Review and Identification of Research Gaps
Source: Microsurgery. 2026 Jan 30;46(2):e70185. doi: 10.1002/micr.70185 (PMC12856973; doi:10.1002/micr.70185)
Supplement: Supplementary file 3 — Supporting Information: 3. ROBINS‐I evaluation of included case‐ control studies. [file MICR-46-e70185-s002.docx]

| **ROBINS-I Domain** | **Bias due to Confounding** | **Selection of Participants** | **Classification of Interventions** | **Deviations from Intended Interventions** | **Missing Data** | **Measurement of Outcomes** | **Selection of Reported Result** | **Overall risk of bias** |
| --- | --- | --- | --- | --- | --- | --- | --- | --- |
| Bauder, 2018, USA* (Bauder et al., 2018) | Serious | Moderate | Low | Moderate | Serious | Moderate | Moderate | Moderate to serious |
|  | *Propensity score matching performed, but residual confounding likely due to small post-bariatric sample and unmeasured variables.* | *Retrospective identification may introduce selection bias based on clinical or referral factors.* | *Exposure (bariatric surgery history) is objective and well-documented.*  *Intervention (DIEP flap after MWL vs DIEP in controls) is objective and unlikely to be misclassified in medical records.* | *Unmeasured variations in perioperative care could differ, though no evidence of systematic deviation.* | *Incomplete follow-up or undocumented minor complications likely; missingness not analytically addressed.* | *Outcomes depend partly on clinician judgment and documentation consistency.* | *No preregistered protocol: selective reporting cannot be excluded.* |  |
| Dayicioglu, 2016, USA* (Dayicioglu et al., 2016) | Serious | Moderate | Low | Moderate | Serious | Moderate | Moderate | Serious |
|  | *Small MWL group (n=6) limits ability to adjust for baseline differences. Important confounders (smoking, diabetes, nutritional status, radiotherapy history, time since weight loss) are likely incompletely measured or unbalanced; even if matching/adjustment was attempted, residual confounding is likely.* | *Retrospective selection may introduce bias (surgeon referral, eligibility for DIEP). The small MWL sample increases the chance that included patients were a selected subset (e.g., healthier or chosen for technical feasibility).* | *Intervention (DIEP flap after MWL vs DIEP in controls) is objective and unlikely to be misclassified in medical records.* | *Perioperative management may vary. But deviations are unlikely to be systematically different in a way that strongly biases the comparison unless care pathways differed between groups.* | *Small sample accentuates impact of any loss to follow-up or incomplete documentation. The study likely relies on chart review; handling of missing outcomes not described.* | *Outcomes such as flap complications, fat necrosis, and revisions are somewhat objective, but some rely on clinician assessment/documentation. Blinding of outcome assessors was not possible, risking differential measurement.* | *No prospective protocol is reported. Risk of selective reporting or emphasis on favourable outcomes exists.* |  |
| Sinik . 2021, USA (Sinik et al., 2023) | Serious | Moderate | Low | Moderate | Moderate | Serious | Moderate | Serious |
|  | *The MWL and non-MWL cohorts differ in baseline BMI (33.0 vs 30.1 kg/m²; P = 0.002) and trend toward more cardiovascular disease. Other important confounders (nutritional deficits, degree of tissue atrophy, prior abdominal surgeries, weight-loss method) are incompletely controlled. The authors use multivariable linear regression (BMI, age, comorbidities), but no matching or propensity methods, and large imbalance in sample size (39 vs 877) increases residual confounding.* | *Retrospective identification of MWL patients from 916 autologous reconstruction cases. Risk of selection bias exists because eligibility for autologous reconstruction may differ for MWL patients (e.g., surgeon selection, patient referral patterns, ability to tolerate long operations). However, all MWL cases during the period were included, suggesting reasonably comprehensive capture.* | *MWL status is defined clearly (>50 lb weight loss or bariatric surgery). Exposure would be correctly recorded in medical charts. Minimal risk of misclassification.* | *Perioperative management was not standardised but likely similar across surgeons at one high-volume academic centre. Still, MWL patients may have received different perioperative optimisation (iron supplementation, nutrition counselling), which could influence outcomes but is unreported. No evidence of deviations caused by knowledge of exposure.* | *As with most retrospective cohorts, documentation variability is likely. BREAST-Q patient-reported outcomes were only returned by 240/425 (56.5%) eligible patients, introducing risk of nonresponse bias. Completeness of late complications is dependent on follow-up; long-term follow-up was not uniformly available. Missingness is unquantified and not handled analytically.* | *Outcomes such as delayed wound healing, surgical-site infection, fat necrosis, and partial flap loss are measured clinically, often based on judgment and documentation. Assessors were not blinded to MWL status. Documentation quality differs across providers. However, many outcomes (e.g., returns to OR, transfusion, flap loss) are objective.* | *No preregistered protocol. Multiple outcomes and subgroup analyses performed; risk of selective reporting exists, although the article provides extensive tables. Regression models are reported selectively (only significant predictors highlighted).* |  |
