# Supplementary material for: Postmastectomy Breast Reconstruction Following Massive Weight Loss: An Updated Systematic Review and Identification of Research Gaps
Source: Microsurgery. 2026 Jan 30;46(2):e70185. doi: 10.1002/micr.70185 (PMC12856973; doi:10.1002/micr.70185)
Supplement: Supplementary file 4 — Supporting Information: 4. Complications and revisions (O1 and O2). [file MICR-46-e70185-s004.docx]

| **Author, year, country** | | **Follow-up (months)** | **Type of reconstruction**  **(no of patients/no of flaps)** | | **Included patients (LWM) (n)**  **(Included breasts (n))** | | | **Included controls (n)**  **(Included breasts (n))** | | **Complications (MWL)** | | **Complications (controls)** | | **Mean revisions (MWL)** | | **Mean revisions (controls)** | | **Comparison between groups**  **(MWL vs. controls)** | | **Comments** |
| --- | --- | --- | --- | --- | --- | --- | --- | --- | --- | --- | --- | --- | --- | --- | --- | --- | --- | --- | --- | --- |
| Case control studies | | | | | | | | | | | | | | | | | | | | |
| Bauder, 2018, USA*(Bauder et al., 2018) (L. Sinik, Egan, Patel, Nazir, & Butterworth, 2022) | 6 | | | Autologous  DIEP (NR/4)  SIEA (NR/5)  TRAM (NR/14)  TUG (NR/3) | | 14  (26) | 1012  (1572)  DIEP (NR/373)  GAP (NR/20)  SIEA (NR/102)  TRAM NR/1053)  TUG (NR/18) | | Fat necrosis: 3 (21%)  Delayed donor site healing: 2 (14%)  Delayed breast healing: 8 (57%) | | Fat necrosis:100 (10%)  Delayed donor site healing: 160 (16%)  Delayed breast healing: 342 (34%) | | Median (IQR)  Revisions: 1.35 (1.15)  Implant/expander placements: 0.42 (0.76)  Total no. of operations: 2.78 (1.36)  8 cosmetic concerns  implant augmentation, lipofilling, mastopexy, Alloderm placement) | | Median (IQR)  Revisions: 0.61 (0.5)  Implant/expander placements: 0.08 (0.41)  Total no- of operations visits: 1.67 (0.90) | | Fat necrosis: p=0.159  Donor site complication: P = 1.00  Delayed breast healing: P = 0.087  Breast revision rate: P = 0.0055  Implant placement procedures: **P =** **0.0003**  The overall number of operations: **P =** **0.0007** | | Complications and revisions are not given separately for different flap types.  Percentages calculated per patient. | |
| Dayicioglu, 2016, USA* (Dayicioglu et al., 2016; L. Sinik et al., 2022) | NR | | | Autologous  DIEP (6/9) | | 6  (9) | 18 | | Flap loss:0  Takeback: 0  Abdominal wound:1  Abdominal hernia:1 | | Flap loss: 2  Takeback: 5  Abdominal wound: 0  Abdominal hernia: 0 | | NR | | NR | | Abdominal bulging or hernia: OR 1.0; 95% CI, 0.078–12.757; P = 1.0  Abdominal wound complications: OR 2.286; 95% CI, 0.266–19.658; P = 0.582 | | Only complications requiring surgery reported  The 18 controls were selected among 97 controls | |
| Sinik . 2021, USA (L. M. Sinik et al., 2023) | 6 | | | Autologous  SIEA (NR/2)  DIEP (NR/60)  PAP (NR/2)  TUG/DUG (NR/4) | | 39 (68) | 877  (139)  SIEA (NR/39)  DIEP (NR/90)  MS-TRAM (NR/14)  PAP (NR/15)  TUG/DUG (NR/75) | | Thrombosis: 1 (1.5%) Congestion: 3 (4.4%) Hematoma: 2 (2.9%) Partial flap loss: 0  Total flap loss: 0 Blood transfusion: 10 (25.6%) Takebacks: 5 (12.8%)  Flap-site complications Delayed wound healing: 16 (23.5%) Surgical-site infection: 5 (7.4%) Seroma: 3 (4.4%) Fat necrosis: 17 (25%)  Partial flap loss: 4 (5.9%) Total flap loss: 0  Donor-site complications  Delayed wound healing:17 (43.6%) Surgical site infection : 0 Seroma 6 (15.4%) Hernia/bulge: 2 (51%) | | Thrombosis: 21 (1.5%) Congestion: 44 (3.2%) Hematoma: 42 (3.0%), Partial flap loss: 8 (0.6%), Total flap loss: 10 (0.7%), Blood transfusion: 86 (9.9%) Takebacks: 85 (9.7%)  Flap-site complications  Delayed wound healing: 163 (11.7%) Surgical-site infection: 31 (2.2%)  Seroma: 40 (2.9%)  Fat necrosis: 224 (16%) Partial flap loss: 22 (1.6%)  Total flap loss: 5 (0.4%)  Donor-site complications  Delayed wound healing:242 (27.6%) Surgical site infection :38 (4.3%) Seroma 6 (15.4%) Hernia/bulge:52 (5.9%) | | Median (IQR) [Mean ± SD]):  No. of fat-grafting revisions: 1 (1) [1.4 ± 1.1]  Fat graft volume (mL per breast): 121.5 (125) [156.7 ± 111.2]  No. of incisional flap revisions: 1 (1) [1 ± 1] vs  No. of donor-site revisions: 1 (1) [0.8 ± 0.8] | | Median (IQR) [Mean ± SD]): No. of fat-grafting revisions: 1 (1) [1.3 ± 1.3] Fat graft volume (mL per breast): 105 (103.1) [174 ± 149]  No. of incisional flap revisions: 1 (1) [1.4 ± 1.1] No. of donor-site revisions: 1 (1) [1.2 ± 1.1] | | Thrombosis: P=0.99 Congestion: P=0.48 Hematoma: P=0.99 Partial flap loss: P=0.99 Total flap loss: P=0.99 Blood transfusion: **P=0.005** Takebacks: P=0.58  Flap-site complications  Delayed wound healing: **P=0.003** Surgical-site infection: **P=0.02** Seroma: P=0.45 Fat necrosis: **P=0.05** Partial flap loss: **P=0.03** Total flap loss: P=0.99  Donor-site complications Delayed wound healing: **P=0.03** Surgical-site infection: P=0.40 Seroma: **P=0.06** Hernia/bulge: P=0.99  Revisions No. of fat-grafting revisions: P=0.73 Fat graft volume (mL per breast): P=0.90 No. of incisional flap revisions: P=**0.009** No. of donor-site revisions: P=**0.01** | | Complications and revisions are not given separately for different flap types.  Percentages calculated per flap, except blood transfusions and takebacks that were calculated per patient | |
| Case series (>10 cases reconstructed with the same technique) | | | | | | | | | | | | | | | | | | |  | |
| Cogliandro, 2018, Italy* (Cogliandro, Barone, Cassotta, Salzillo, & Persichetti, 2018; L. Sinik et al., 2022) | 12 (range 4-36) | | | Implant-based | | 20 | NA | | 1 wound dehiscence  1 asymmetry | | NA | | NR | | NA | | NA | |  | |
| Martinez, 2016, USA (Martinez, Walters, Sato, Hall, & Boutros, 2016; L. Sinik et al., 2022) | 16.2 (range 3.5 to 28.7) | | | Autologous  DIEP | | 9 (18) | NA | | 0 | | Flap loss: 0 Breast abscess: 1  Abdominal suture cyst: 1 | | NA | | 0 | | NA | | 13 immediate and 5 delayed cases | |
| Case reports (<10 cases reconstructed with the same technique | | | | | | | | | | | | | | | | | | |  | |
| Abdel-Naby, 2017, USA* (Abdel-Naby, Ablavsky, & Shteynberg, 2017; L. Sinik et al., 2022) | 2 | | | Autologous  TRAM | | 1 | NA | | Bilateral Sciatic neuropathy | | NA | | NR | | NA | | NA | |  | |
| Asiry, 2019, France* (Asiry, Garrido, Chaput, Chantalat, & Vaysse, 2019; L. Sinik et al., 2022) | 12 | | | Reverse abdominoplasty advancement+ lipofilling | | 1 | NA | | None | | NA | | 1 (lipofilling, inframammary fold revision) | | NA | | NA | |  | |
| Berkane . 2024, USA (Berkane et al., 2024) | 6 | | | Autologous  Fleur-de-lis DIEP | | 1 | NA | | None | | NA | | None | | NA | | NA | |  | |
| Chakari . 2024, Denmark (Chakari, Bille, Lilja, & Thomsen, 2024) | NR | | | Autologous  Stacked IMAP+TDAP  Stacked TDAP+SEAP | | 2 | NA | | NR | | NA | | NR | | NA | | NA | |  | |
| Gusenoff, 2009, USA*(Gusenoff et al., 2009) (L. Sinik et al., 2022) | 18 | | | Autologous  TRAM (1/4)  DIEP (1/1) | | 3  (5) | NA | | Masteoctmy flap necrosis (TRAM): 1  Abdominal bulge (TRAM): 1  Lipoma in reconstructed breast (DIEP): 1 | | NA | | NR | | NA | | NA | |  | |
| Salim, 2013, UK* (Salim, Adlard, & Pickford, 2013; L. Sinik et al., 2022) | 6 | | | Autologous  Fler de lis DIEP | | 1 (1) | NA | | Abdominal seroma: 1 | | NA | | 1 (correction of asymmetry) | | NA | | NA | |  | |
| Söderman, 2021, Denmark (Soderman, Thomsen, & Sorensen, 2021) | 3 | | | Autologous  VMG | | 1 (1) | NA | | Haematoma donor site: 1 | | NA | | 1 (contralateral thigh plasty+laser hair removal) | | NA | | NA | |  | |
| Wechselberger, 2000, Switzerland* (L. Sinik et al., 2022; Wechselberger, Haug, Schoeller, Nehoda, & Piza-Katzer, 2000) | NR | | | Autologous  DIEP | | 1 (2) | NA | | None | | NA | | None | | NA | | NA | |  | |
| Yoo, 2022, USA (Yoo et al., 2022) | 7 | | | Autologous  Stacked DIEP+ vPAP | | 2 (4) | NA | | Fat necrosis abdomen: 1 | | NA | | Wise pattern skin reduction: 1  Lipofilling: 2  Abdominal scar revision: 2  Dog ears excised from the thighs: 2 | | NA | | NA | | 1 delayed and 1 immediate case  TEs were placed before autologous reconstruction in the delayed case  A dermal sling was used in the immediate case | |

BMI body mass index

CI confidence interval

DIEP Deep Inferior Epigastric Perforator (flap)

DUG Diagonal Upper Gracilis (flap)

GAP  Gluteal Artery Perforator (flap)

IMAP Internal Mammary Artery Perforator (flap)

LD latissimus dorsi (flap)

MWL massive weight loss

N number

NA not applicable

NR not reported

MS muscle sparing

OR odds ratio

PAP Profunda Artery Perforator (flap)

SD standard deviation

SEAP Superficial External Abdominal Perforator (flap)

SIEA Superficial Inferior Epigastric Artery (flap)

TDAP Thoracodorsal Artery Perforator (flap)

TRAM Transverse Rectus Abdominis Myocutaneous (flap)

TUG/DUG Transverse Upper Gracilis (flap)/

V vertical

VMG vertical gracilis myocutaneous flap
